# Supplementary material for: Measuring Heart Rate Variability in Patients Admitted with ST-Elevation Myocardial Infarction for the Prediction of Subsequent Cardiovascular Events: A Systematic Review
Source: Medicina (Kaunas). 2021 Sep 26;57(10):1021. doi: 10.3390/medicina57101021 (PMC8540987; doi:10.3390/medicina57101021)
Supplement: Supplementary file 1 [file medicina-57-01021-s001.zip › Table S1 Databases and search strategy.pdf]

Table S1. Databases and search strategies used in present systematic review

Database	Coverage	Search run	Records
**MEDLINE**	1946 to present	"Heart rate variability AND myocardial infarction"	457
		"heart rate variability AND ST-elevation myocardial infarction"	43
		"heart rate variability AND myocardial infarction AND cardiovascular events"	78
		"heart rate variability AND myocardial infarction AND risk assessment"	82
		"heart rate variability AND myocardial infarction AND mortality"	243
			Total records = 903
**Embase**	1966 to present	"Heart rate variability AND myocardial infarction"	243
		"heart rate variability AND ST-elevation myocardial infarction"	25
		"heart rate variability AND myocardial infarction AND cardiovascular events"	48
		"heart rate variability AND myocardial infarction AND risk assessment"	64
		"heart rate variability AND myocardial infarction AND mortality"	0
			Total records = 380
**Cochrane library**	1967 to present	"Heart rate variability AND myocardial infarction"	823
		"heart rate variability AND ST-elevation myocardial infarction"	53
		"heart rate variability AND myocardial infarction AND cardiovascular events"	191
		"heart rate variability AND myocardial infarction AND risk assessment"	277
		"heart rate variability AND myocardial infarction AND mortality"	365
			Total records = 1709
