# Supplementary material for: Measuring Heart Rate Variability in Patients Admitted with ST-Elevation Myocardial Infarction for the Prediction of Subsequent Cardiovascular Events: A Systematic Review
Source: Medicina (Kaunas). 2021 Sep 26;57(10):1021. doi: 10.3390/medicina57101021 (PMC8540987; doi:10.3390/medicina57101021)
Supplement: Supplementary file 1 [file medicina-57-01021-s001.zip › Table S2 General characteristics.pdf]

Table S2. General characteristics of studies included in present systematic review.

Author, year	Design	Patients, No	Age median/ mean ± SD	Parameters evaluated	Setting	Outcomes	Timing and methods
Balanescu et al, 2004	Observational, prospective	463	60.6±13.0	SDNN<br>RMSSD<br>LF<br>HF<br>LF/HF<br>Total spectral power	Patients with STEMI in the first 24 hours from chest pain onset treated with thrombolysis (n=133, 28.7%), pPCI (n=79, 17.1%) in the first 12 hours (n=211) or with conventional therapy (n=251, 54.2%), with 1-year follow-up period	*Primary endpoint*: 1-year vital status (sudden cardiac death, cardiac and extra-cardiac death)<br><br>*Secondary endpoints*: angina recurrence, MI or heart failure symptoms	24-hour Holter ECG recording at 10-20 days after MI
Boskovic et al, 2014	Observational, prospective	100	56.99±11.03	SDNN<br>Mean RR interval<br>RRmax-RRmin	Patients with STEMI treated with thrombolysis, pPCI or medical treatment and 1-year follow-up	All-cause mortality	24-hour Holter ECG recording at 8-13 days after MI
Chakrovortty et al, 2011	Cross-sectional	105	53.9±10.8 (male)<br>59.8±8.8 (female)	SDNN<br>Mean RR interval	Patients with STEMI treated mainly with thrombolysis (n=103)	Correlation between HRV and TIMI risk score	24-hour Holter ECG recording during hospitalization
Compostella et al, 2017	Observational, retrospective	208 (STEMI)	61.3±12.5	SDNN<br>SDANN<br>RMSSD<br>SDNNi	Patients with STEMI, with 94% successful PCI rate (n=194) within 6 hours from	*Primary outcome*: cardiac death	24-hour Holter ECG recording after 13.5 days from index

		118 (NSTEMI)	67.4±10.4		symptoms onset and 25 months median follow-up period	*Secondary end point*: major clinical events (all-cause and cardiac mortality, readmission for a new MI, repeated revascularization, heart failure, stroke)	event (when patients were admitted to cardiac rehabilitation unit)
Coviello et al, 2013	Observational, prospective	182	59.1±11	Mean RR interval<br>SDNN<br>SDNNi<br>VLF<br>LF<br>HF	Consecutive patients with STEMI treated by pPCI or rescue PCI and optimal medical therapy with 42±23 months follow-up period	Major clinical events defined as death or reinfarction	24-hour Holter ECG recording before discharge (after 3-10 days from the event) and at 1 and 6 months after discharge
Ablonskyte-Dudoniene et al, 2012	Observational, prospective	213	63 (53-70)	SDNN<br>RMSSD	Non-consecutive patients with STEMI with symptoms onset <24 hours, treated mainly by PCI (89.9%), with follow-up at 1-year and 5-years	All-cause mortality<br>Cardiac mortality	24-hour Holter ECG recording on day 1 and day 3 after MI
Erdogan et al, 2008	Observational, retrospective	412	60±12	SDNN<br>RMSSD	Unselected patients with STEMI treated with pPCI within 12 hours from symptoms onset, with 4.3±3 years follow-up period	All-cause mortality<br>Cardiac mortality	24-hour Holter ECG recording after 11±9 days from the index MI
Karp et al, 2009	Observational, retrospective	196	60.9±13	SDNN	Consecutive patients with STEMI, treated	All-cause mortality<br>CABG	10-second ECG recording

					with fibrinolysis or pPCI, with 2 years follow-up period	Reinfarction<br>Cardiac admission	prior to reperfusion therapy and at discharge
Katz et al, 1999	Observational, prospective	185	56±11	RRmin-RRmax<br>SDANN	Consecutive STEMI patients treated with thrombolysis (64.4%) or conventional medical therapy (35.6%) and 16 months mean follow-up period	Adverse cardiac events (MI, congestive heart failure, angina, death)<br>Recurrent MI<br>All-cause mortality	1-minute ECG recording in deep breathing at 5.1±2.5 days after the index event

CABG = coronary artery bypass graft; ECG = electrocardiography; HF = power in high frequency range; HRV = heart rate variability; LF = power in low frequency range; MI = myocardial infarction; NSTEMI = Non-ST-Elevation Myocardial Infarction; PCI = percutaneous coronary intervention; pPCI = primary percutaneous coronary intervention; RMSSD = the square root of the mean of the sum of the squares of differences between adjacent NN intervals; RRmax-RRmin = difference between the longest RR interval and the shortest RR interval; SDANN = standard deviation of the averages of NN intervals in all 5 min segments of the entire recording; SDNN = standard deviation of all NN intervals; SDNNi = mean of the standard deviations of all NN intervals for all 5 min segments of the entire recording; STEMI = ST-elevation myocardial infarction; TIMI = The Thrombolysis in Myocardial Infarction; VLF = power in very low frequency range.
