# Supplementary figures and images for: Measuring Heart Rate Variability in Patients Admitted with ST-Elevation Myocardial Infarction for the Prediction of Subsequent Cardiovascular Events: A Systematic Review
Source: Medicina (Kaunas). 2021 Sep 26;57(10):1021. doi: 10.3390/medicina57101021 (PMC8540987; doi:10.3390/medicina57101021)

**Figure S1.** Flow diagram of selected studies in present systematic review.

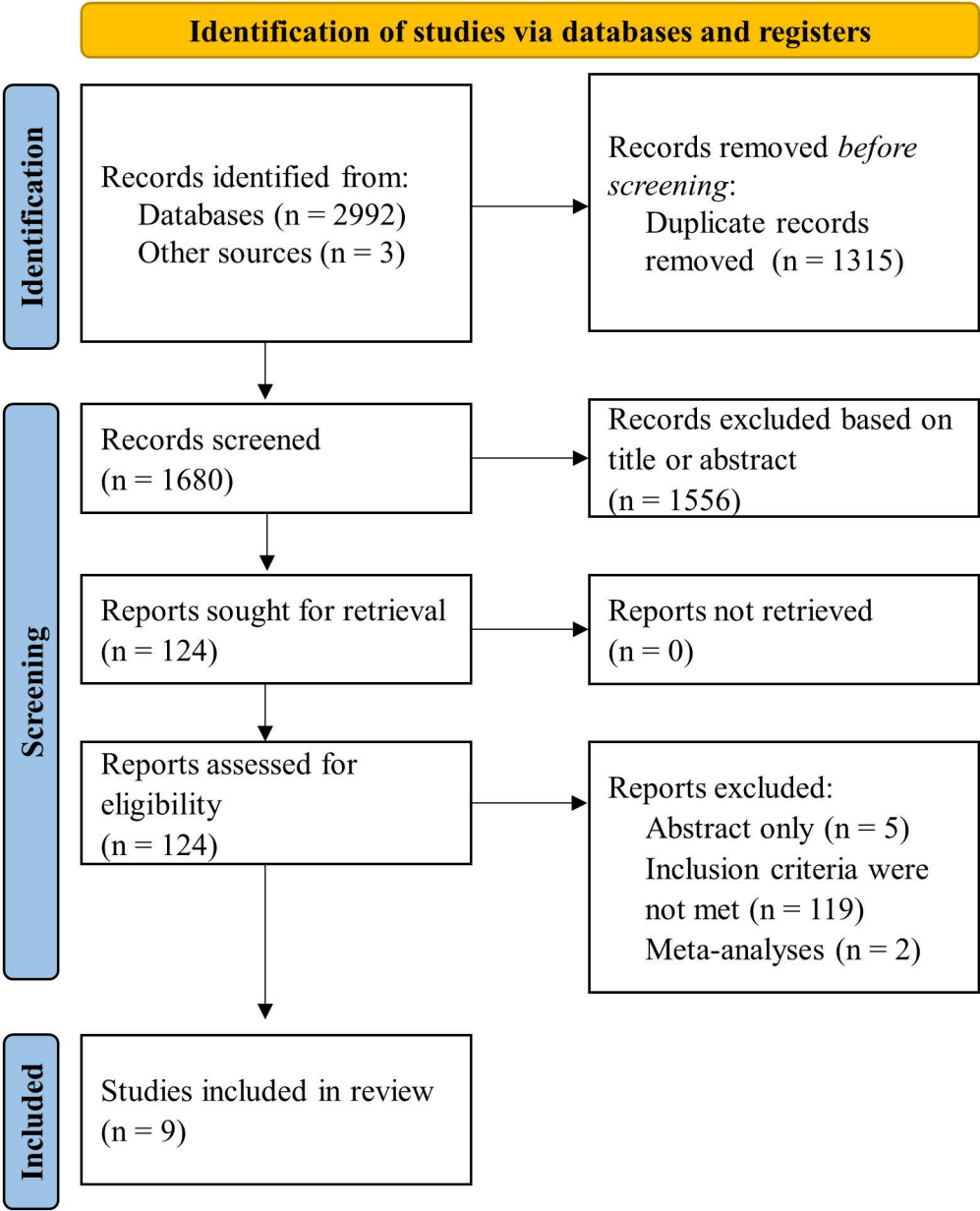

Supplement: Supplementary file 1 [file medicina-57-01021-s001.zip › Figure S1. PRISMA flow diagram ed.pdf]
